# Negative effects in psychotherapy: commentary and recommendations for future research and clinical practice

**DOI:** 10.1192/bjo.2018.42

**Published:** 2018-07-25

**Authors:** Alexander Rozental, Louis Castonguay, Sona Dimidjian, Michael Lambert, Roz Shafran, Gerhard Andersson, Per Carlbring

**Affiliations:** Researcher, Centre for Psychiatry Research, Department of Clinical Neuroscience, Karolinska Institute, Sweden and Institute of Child Health, University College London, UK; Professor, Department of Psychology, Penn State University, USA; Associate Professor, Department of Psychology and Neuroscience, University of Colorado Boulder, USA; Professor, Department of Psychology, Brigham Young University, USA; Professor, Institute of Child Health, University College London, UK; Professor, Department of Behavioural Sciences and Learning, Linköping University and Centre for Psychiatry Research, Department of Clinical Neuroscience, Karolinska Institute, Sweden; Professor, Department of Psychology, Stockholm University, Sweden and Department of Psychology, University of Southern Denmark, Denmark

**Keywords:** Negative effects, psychotherapy, SWOT, commentary

## Abstract

**Background:**

Psychotherapy can alleviate mental distress and improve quality of life, but little is known about its potential negative effects and how to determine their frequency.

**Aims:**

To present a commentary on the current understanding and future research directions of negative effects in psychotherapy.

**Method:**

An anonymous survey was distributed to a select group of researchers, using an analytical framework known as strengths, weaknesses, opportunities and threats.

**Results:**

The researchers perceive an increased awareness of negative effects in psychotherapy in recent years, but also discuss some of the unresolved issues in relation to their definition, assessment and reporting. Qualitative methods and naturalistic designs are regarded as important to pursue, although a number of obstacles to using such methods are identified.

**Conclusion:**

Negative effects of psychotherapy are multifaceted, warranting careful considerations in order for them to be monitored and reported in research settings and routine care.

**Declaration of interest:**

None.

Psychotherapy has the potential of alleviating mental distress and improving quality of life for many patients. However, despite promising research results and an increased dissemination of effective treatments, little is known about their potential negative effects. Possible harmful events have been explored, both in terms of deterioration of symptomatology and adverse and unwanted events, but more needs to be done with respect to understanding, monitoring and reporting their incidents. This article discusses some of the current findings and future challenges with regard to investigating and reporting negative effects of psychotherapy.

Negative effects belong to a relatively unchartered territory of psychotherapy research, as little attention has been given to the possibility that some patients might deteriorate or experience adverse or unwanted events during treatment.^1^ Two recent editorials and a cross-sectional survey published in the *British Journal of Psychiatry* have recognised the need for further study of negative effects, and provided empirical evidence of their incidence among patients receiving psychological treatment through the National Health Service in the UK.^2^^–^^4^ In total, 5.2% of the respondents stated that they had experienced lasting negative effects, and findings indicated that the odds of such events were higher among minority groups and those who were uncertain about what type of treatment they had received. Recommendations on how to advance the research on negative effects were also put forward, such as, the use of a more coherent terminology, clearer procedures for monitoring and reporting negative effects, and notifying patients of the potential risks of treatment during the informed consent process.

We sought to address these recommendations by administering a survey to a select group of researchers in the field of psychotherapy. The aim was to provide an overview of expert perceptions of what is known of the occurrence and characteristics of negative effects and how well they are currently being studied. In addition, the purpose was to summarise expert recommendations regarding strategies to better understand and investigate negative effects, primarily in a research setting. In so doing, this study may contribute to efforts in raising awareness of such events among researchers and clinicians, improve their monitoring and reporting in routine care and research settings, and shed some light on the complex nature of exploring negative effects in psychotherapy.

## Method

A select group of researchers in the field of psychotherapy were invited to share their perspective on the topic of negative effects. These were chosen on the basis of prior experience in psychotherapy research, in particular, investigating events that can be considered harmful, for example deterioration, either through prior publications or reputation. In this sense, the recruitment to the group can be regarded as convenience sampling. Of the ten researchers that were invited, two declined participation because of time constraints, and one did not respond despite several reminders. Hence, included in the commentary were seven respondents; two women and five men, with three being from Sweden, one from the UK and three from the USA. Given that no patient data was included, no ethics approval was warranted.

A survey was administered online using an analytical framework known as strengths, weaknesses, opportunities and threats (SWOT). Pickton & Wright^5^ described this as ‘the collection and portrayal of information about internal and external factors which have, or may have, an impact on business’, that is, identifying the resources, limitations, possibilities and risks of a particular area of interest. Most often, SWOT is applied in organisational or financial settings to enhance creativity and predict upcoming competition. However, this framework can also be used in domains unrelated to any commercial interest, such as, determining the usefulness and drawbacks of a new intervention, for example virtual reality therapy.^6^ The researchers were thus encouraged to give their opinion on how the investigation of negative effects in psychotherapy is being performed and might be enhanced, using the guidelines in SWOT to facilitate a discussion (see the Appendix). The survey was completely anonymously in order to reduce risk of conformity and social inhibition. A.R. then compiled a summary that was sent out to everyone involved in order for them to comment on each other's responses, also anonymously. The responses were examined thematically to derive recurrent themes. Finally, a draft of the results was prepared and examined by the researchers before being submitted for potential publication. This permitted the provision of additional information throughout the process and allowed revisions to be made in relation to previous statements. A similar approach has been applied for developing a consensus of monitoring and reporting negative effects in psychotherapy delivered via the internet.^7^

In the current commentary, the term negative effects is used to describe potentially harmful events more globally, as proposed by Hadley & Strupp,^8^ that is, including not only deterioration in symptomatology, but also other potential adverse and unwanted events. Meanwhile, deterioration refers to the change between two points of measurement in a negative direction, which is often determined using the Reliable Change Index (RCI).^9^ Adverse and unwanted events are used to describe those situations where the patient experiences an unforeseen and undesirable effect, which does not have to be associated with increased symptomatology, for example novel symptoms and stigma.^10^

## Results

### Strengths

#### Greater awareness of negative effects

The awareness of patients experiencing negative effects in psychotherapy is believed to have improved during the past decade. In particular, clinicians and researchers have become more mindful of the fact that a proportion of patients deteriorate during treatment, and that there are reliable methods to detect and monitor patients where improvement is absent. However, it is clear that clinical training rarely includes information about negative effects,^11^ and that researchers seldom report negative effects in clinical trials.^12^ Also, the use of practices for assessing deterioration is still missing in most contexts, despite evidence suggesting that the rate of deterioration can be reduced from 23.2 to 15.2% just by using between-session monitoring,^13^ and be as low as 8.5% by also providing the clinician with tools for problem-solving.^14^ Moreover, reporting negative effects of psychotherapy is still not mandatory in most scientific journals, making it difficult to determine their occurrence and characteristics. Thus, although the respondents reported their perceptions of recent improvements in awareness, they also stated that much more needs to be done in order to improve both research and clinical practice in terms of detecting, reporting, and averting negative effects.

#### Progress monitoring

Deterioration is difficult to distinguish based on clinical judgement alone, and evidence suggests that clinicians are often poor at recognising if a patient has worsened.^15^ Introducing some form of progress monitoring has therefore been proposed as a more accurate way of identifying those who do not improve, with several studies providing evidence for its superiority.^16^ Basically, progress monitoring employs statistical and actuarial procedures to determine if a patient deviates from an expected trajectory. If that is the case, the clinician or researcher will be notified in order to ensure that the correct actions are implemented, for example considering other treatment alternatives. This type of notification has not been found to boost effects or cut costs, rather, the advantages seem to be lower rates of deterioration and non-response among patients receiving treatment.^17^ Using data from progress monitoring could also make it possible to explore what factors might be responsible for worsening and lack of change. This should, in turn, help improve how psychotherapy is being delivered as well as increase the understanding of what actually constitutes functionality and dysfunctionality with regard to a patient's mental health.

#### Qualitative methods

An increase in existing symptoms (i.e. deterioration) is probably the most straightforward way of exploring negative effects of psychotherapy. However, different qualitative methods for investigating other harmful events also exist. Interviews or self-report measures may facilitate an in-depth exploration of detrimental effects, such as asking patients to describe negative experiences during treatment, similar to the use of open-ended questions in a recent study of cognitive–behavioural therapy delivered via the internet.^18^ Furthermore, several instruments for assessing potentially harmful events during treatment have recently been proposed, such as, the Negative Effects Questionnaire,^10^ which could be useful for determining the relationship between adverse and unwanted events and treatment outcome. Presently, qualitative methods are seldom used in relation to negative effects but have been put forward as a way of improving the understanding of their occurrence and characteristics.^19^

### Weaknesses

#### Definitions

Negative effects of psychotherapy presently lack a clear and coherent terminology, possibly restricting the monitoring and reporting of their incidence. Various propositions with regard to definitions and consistency of terms have been proposed,^7^^,^^19^^,^^20^ but no consensus currently exists as to what events need to be explored. Most terms involve the exacerbation of symptoms or new forms of distress, but incidents of malpractice might also be important to consider. In addition, it is not evident what distinguishes some negative effects, such as, deterioration, from non-response or unmet treatment expectations, which might be just as detrimental to the patient. Furthermore, even in relation to worsening, which relies on a statistical procedure such as the RCI, it not yet apparent if this corresponds to a negative experience that happened during or was caused by psychotherapy.^21^ Also, ceiling effects prevent patients from deteriorating indefinitely during treatment. Although a recent individual patient meta-analysis found that only 1.9% of 2866 patients were close to hitting the ceiling, there might be particular patient groups where this risk is much higher.^22^ Likewise, increase in symptomatology may reflect deterioration within one domain, but it has been suggested that both improvement and worsening could be made up of several distinct areas of functioning, such as interpersonal problems, quality of life, and family distress.^23^ Hence, several issues need to be addressed in the future in order to improve the monitoring and reporting of negative effects, both in terms of their definitions and clinical significance.

#### Time period

The study of negative effects is highly influenced by the time period during which they are being evaluated. However, deterioration and other potentially harmful events have mostly been determined on one single occasion, making it unclear if they are transient or enduring. Many patients may experience situations in treatment as negative because they result in unwanted feelings or thoughts that have previously been avoided, even though it might be beneficial in the long run, such as gradual exposure to certain stimuli in anxiety disorders.^24^ Thus, without the use of a follow-up assessment it is uncertain how incidents such as deterioration are associated with long-term treatment outcome.^22^ Also, investigating other types of negative effects solely after treatment has ended, for instance, by distributing open-ended questions or self-report measures, are highly susceptible to the effects of memory bias and social desirability, affecting the validity of the responses. Exploring negative effects at different time points is therefore important to consider but has so far not warranted sufficient attention among clinicians or researchers.

#### Perspective

Determining whether negative effects have occurred during treatment largely depends on what perspective is endorsed. Incidents might, for instance, be considered adverse and unwanted by a patient, but deemed an inevitable part of certain interventions by a clinician. Even results that are regarded as positive may potentially be viewed as detrimental by others, as in the case of someone who becomes more self-assertive towards relatives or co-workers. There may also be a discrepancy between achieving good results in psychotherapy but still not being satisfied by treatment outcome. The idea that the benefits in psychotherapy could be perceived differently by the patient and significant others is nothing new,^25^ but could be particularly important in relation to negative effects as it is unclear if their incidents can be attributed to treatment or other circumstances in the patients' lives. This might therefore warrant the implementation of different perspectives in research and clinical practice, such as considering various types of information on progress.^26^ For instance, the respondents discussed the possibility of exploring the positive as well as negative effects of psychotherapy by interviewing significant others, comparing clinician ratings to patient self-reports, and by distributing an instrument specifically on negative effects to several parties. This might, however, only be possible in certain research settings where the patients consent to this type of investigation, and less so in clinical practice.

#### Reporting

Negative effects are increasingly being reported in peer-reviewed journals, even though far from every clinical trial includes this information.^12^ However, the presentation of such incidents is not always optimal, seldom including any details of how deterioration or adverse or unwanted events were assessed. Editors therefore need to become more aware of how negative effects can be monitored and reported, and, possibly, also making it mandatory for researchers to include data of their occurrence as secondary analyses, similar to exploring moderators of treatment outcome. Given the limited space in peer-reviewed journals, it is understandable that the investigation of negative effects is often left out, but alternatives for their inclusion and distribution should be possible. For instance, by providing supplementary material online, or, as in the case of studies funded by the National Health Service in the UK, including a monitoring system that can end a treatment prematurely if harm is observed.

### Opportunities

#### Effective methods

Promising methods for monitoring and preventing negative effects already exist, such as progress monitoring by distributing self-report measures at each session. This has been shown to improve the detection of deterioration, which can help to reverse a negative treatment trend and minimise harm in psychotherapy.^16^ Additional research needs to be performed in more settings and across different samples in order to establish its benefits in clinical practice. Particularly, studies need to be done in larger samples to increase statistical power and preferably where the feedback measure is different from the one assessing treatment outcome. However, this is only relevant for studying deterioration, as adverse and unwanted events cannot be captured by this method. Yet, given the ease of implementation and its possibility to determine worsening, progress monitoring could be seen as a first, but not the only step in making the assessment and investigation of negative effects an integral part of clinical practice and research.

#### Informed consent

Based on the current understanding, information about both the expected results and potential negative effects should be conveyed to all patients before commencing treatment. Thereby, the risk of deterioration and experiencing adverse and unwanted events can be discussed carefully, possibly preventing or at least minimising the impact of such events. Moreover, addressing the issue of negative effects early on could also help explore the expectations or idiosyncratic beliefs the patient can have, such as, not wanting to experience uncomfortable feelings or having unrealistic ideas about treatment outcome. Both the pros and cons of treatment should thus be reviewed, allowing the patient to make an informed decision about whether to pursue psychotherapy or implement specific interventions. Although it has been shown that clinicians and researchers are reluctant to mention possible risks of undergoing treatment,^27^ it has not yet been established empirically that such information induces harm or affects the therapeutic relationship. Providing the patient with all necessary details regarding both the advantages and disadvantages of undergoing psychotherapy should therefore always be addressed during the informed consent process.

#### Naturalistic designs

Negative effects are often explored within the context of clinical trials that are performed in highly controlled settings. Even if this is important to increase internal validity, it is also necessary to investigate harmful events in routine care. Practice-oriented research conducted in the natural environment of the clinician could potentially yield valuable information regarding the occurrence and characteristics of deterioration, non-response and adverse and unwanted events among patients receiving psychotherapy in a clinical setting. For instance, a recent naturalistic study indicated that more than half of the patients did not change reliably, and that a larger proportion deteriorated in a psychiatric context (6.9%) compared with primary care (1.8%).^28^ Using data from such research could help pinpoint certain interventions as harmful and how it has an impact on the therapeutic relationship, as well as facilitate an investigation of how the occurrence of negative effects differ between clinicians, such as, a higher probability of deterioration among patients in comparison with colleagues.^29^

### Threats

#### Reluctance

Despite many advantages of assessing negative effects in psychotherapy, clinicians and researchers are often reluctant to implement effective methods. In particular, systems for tracking patients' status (i.e. progress monitoring) might be perceived as threating as the information is forwarded to the treatment provider, increasing accountability. Being unaware of how such information is used could make many clinicians insecure of its implementation, especially if it is used to compare colleagues with each other or to blame an inferior treatment outcome on a specific clinician. In the long run this might lead to a situation where the more severe patients are referred elsewhere. Also, the costs of introducing monitoring systems may inhibit its administration, both in terms of the money required to set it up in routine care and research settings, and the time it will require for clinicians and researchers to adjust to new procedures and input relevant data.

#### Lack of knowledge

Clinicians and researchers are becoming increasingly aware of the issue of negative effects in psychotherapy. Nonetheless, far from everyone knows that there exist potential risks and not all seem to agree on their characteristics.^30^ In addition, it can also be assumed that some might even perceive negative effects as necessary in order to produce a positive treatment outcome (i.e. the need for the patient to feel worse before feeling better). However, disseminating research on negative effects in routine care is difficult given the workload in most settings, and there are countries where it is not required by clinicians to be updated on the latest findings as part of their professional practice. Furthermore, many researchers are still unaware of detrimental effects in psychotherapy despite the fact that it has been the topic of great debate for many years. Lack of knowledge concerning negative effects therefore poses one of the greatest threats to the monitoring and reporting of their incidence.

#### Expectations

Investigating and informing about negative effects is considered important. However, there is also a possibility that it could affect the expectations of the patient. By putting too much emphasis on the risks of psychotherapy this might interfere with the expectations of getting better by undergoing treatment. In addition, focusing on reducing experiences that can be perceived as negative by the patient could potentially also interfere with certain parts of the interventions that are in fact beneficial (i.e. avoiding the confrontation of specific themes). Moreover, using the same researcher to investigate both the positive and negative effects might introduce some bias, for example the risk of patients' not reporting harm in an otherwise successful treatment, which should warrant methodological considerations as to how the implications of psychotherapy are explored.

## Discussion

In this study we have summarised current understanding and identified future research directions of monitoring and reporting of the negative effects of psychotherapy by asking a select group of researchers in the field to share their perceptions of these topics. Everyone agreed that the awareness of the risks involved in treatment have increased recently. However, a number of obstacles were also proposed, including the lack of a clear and coherent terminology, methodological issues and problems related to reporting negative effects in many scientific journals. These results are in line with the two recent editorials and a cross-sectional survey published in the *British Journal of Psychiatry*,^2^^–^^4^ suggesting that more needs to be done with regard to how clinicians and researchers study and report events in treatment that are negative. Particularly, a consensus on definitions would be valuable, as it might lead to a more systematic approach to exploring negative effects of psychotherapy (i.e. assessing the same type of incidents). Furthermore, several aspects related to the methodology being used also need to be agreed upon, for example, the time period that deterioration should be studied to determine if it is transient or enduring, and what perspectives to consider, for example the patient, clinician or significant others. Using naturalistic designs and qualitative methods could also prove to be important, as it might reveal factors related to worsening and adverse and unwanted events, as well as how patients themselves perceive potentially harmful events in psychotherapy. Meanwhile, both clinicians and researchers need to be informed about the possibility that treatments are not without risks, especially since prior evidence implies that clinical training seldom includes information about negative effects.^11^ This could become an integral part of their training, similar to teaching the basics of a particular therapeutic orientation or the ethics and jurisdiction involved in providing healthcare. In addition, informing patients about the benefits as well as risks of psychotherapy should be seen as mandatory during the informed consent process, allowing patients to make an educated decision on whether or not to pursue treatment or implement certain interventions.

As for research practices, editors of scientific journals could demand the reporting of negative effects prior to a potential publication, either as secondary analyses included in a manuscript or accessible as supplementary material offered online. Given the lack of consensus about how to define potentially harmful events, using the RCI to determine reliable deterioration of symptomatology could be a possible first step, as well as the inclusion of open-ended questions regarding negative experiences among the patients. Finally, progress monitoring is regarded as an effective method for preventing deterioration and should become more widely used in routine care as well as research settings.

However, a number of issues need to be resolved with regard to accountability and how the information such a procedure provides is going to be used, for example becoming liable for patient worsening. Furthermore, apart from an increase in symptomatology, other dimensions of functionality may be important to consider in relation to treatment outcome, for instance, more global measures of well-being. Nonetheless, monitoring treatment progress on a session-by-session basis is seen as a promising way of minimising the risk of deterioration in psychotherapy, as well as being useful for the exploration of likely mechanisms responsible for negative effects.

### Limitations

The present commentary summarises the results and experiences of investigating negative effects in psychotherapy made by a select group of researchers in the field. As such, it provides valuable insights with regard to the many methodological issues and practical difficulties involved in monitoring and reporting potentially harmful events in treatment. However, given the lack of a systematic approach and recruiting a limited number of researchers in the field the results that have been presented should not be seen as exhaustive or an attempt to cover all aspects on this topic. In addition, by not including clinicians, there is also a risk of missing important aspects related to negative effects in psychotherapy, such as working with particular patient groups. Therefore, additional perspectives on the subject of negative effects of psychotherapy most certainly exist and might have emphasised a different set of issues than those that have been put forward, limiting the generalizability of the results. Furthermore, the use of an anonymous survey may perhaps have prevented conformity and social inhibition, allowing the researchers to more freely express their opinions and comment on each other. However, given the novelty of administering SWOT for exploring a particular topic in psychotherapy, it is unknown if other means would have revealed different results. Similarly, given the approach used, the results were not coded or analysed according to the steps usually employed in, for example, thematic analysis. This makes the issues of credibility and transferability as referred to in qualitative studies less clear, and should be regarded as a limitation when interpreting the results. The conclusions should therefore by no means be seen as definitive, but rather as a reflection of how negative effects are currently being researched and how it can be improved further in the future.

## Appendix

### Survey

Write your response to the four SWOT questions below. The survey is anonymous. You can be as brief or elaborate as you wish, using the concepts of Strengths, Weaknesses, Opportunities and Threats in relation to negative effects of psychological treatments.

1. Strengths: What are we doing well in terms of investigating the occurrence and characteristics of negative effects? What resources can we draw on? Strengths can refer to quality and reliability, reflecting aspects to capitalise on, e.g., ‘Using the Reliable Change Index as a measure of deterioration in clinical trials’.
2. Weaknesses: What could we improve with regard to examining the occurrence and characteristics of negative effects? What factors might be seen as limitations? Weaknesses can refer to absence of strengths or aspects that may be turned into strengths or need to be solved, e.g., ‘Inconsistencies in reporting deterioration rates and adverse events in clinical trials’.
3. Opportunities: What opportunities are currently open in studying the occurrence and characteristics of negative effects? What trends could we take advantage of? Are there any identified strengths that we could turn into opportunities? Opportunities can refer to needs that are not yet addressed or must be acted upon, e.g., ‘Systematically collecting patient-level data in clinical trials to facilitate an inspection of predictors for deterioration across psychological treatments’.
4. Threats: What threats could limit or become an obstacle for researching the occurrence and characteristics of negative effects? Are there any identified weaknesses that might become threats? Threats can refer to events or forces that need to be planned for, e.g., ‘Lack of knowledge among clinicians and researchers with regard to monitoring and reporting negative effects of psychological treatments’.
